# Growth mechanism of SnC_2_H_4_O_2_ nanowires prepared by the polyol process as SnO_2_ precursor nanowires†

**DOI:** 10.1039/c8ra09738k

**Published:** 2019-01-23

**Authors:** DongKook Park, Man Sig Lee

**Affiliations:** Green Materials & Processes Group, Korea Institute of Industrial Technology (KITECH) 55, Jongga-ro, Jung-gu Ulsan Republic of Korea lms5440@kitech.re.kr

## Abstract

Tin oxide (SnO_2_) nanowires are produced by the calcination of tin glycolate (SnC_2_H_4_O_2_) nanowires, which are synthesized with tin oxalate (SnC_2_O_4_) and ethylene glycol *via* the so-called polyol process. In this study, the growth mechanism of SnC_2_H_4_O_2_ nanowires was investigated by monitoring the synthesis using scanning and transmission electron microscopy. The length and diameter of the nanowires were 9.25 μm and 0.37 μm, respectively; the former increased at a rate of 1.85 μm h^−1^ but the latter did not increase over time. Fourier-transform IR spectroscopy showed that the nanowires were composed of SnC_2_H_4_O_2_ instead of SnC_2_O_4_. Changes in the components of the reaction solution were also confirmed by ^1^H NMR, ^13^C NMR, and high-performance liquid chromatography. SnC_2_H_4_O_2_ was formed by the substitution of the oxalate coordinated to tin by ethylene glycolate, which was produced by the deprotonation of ethylene glycol. In this reaction, oxalate gradually changed to formic acid and carbon dioxide, and SnC_2_H_4_O_2_ grew as a nanowire through O–Sn–O bond formation. In addition, when ethylene glycol was mixed with 1,2-propanediol, branched SnC_2_H_4_O_2_ nanowires were formed. The branching was due to the interference of the methyl group of 1,2-propanediol with the growth of bundle-type nanowires. The branched nanowires had a higher surface area-to-mass ratio than the bundled ones based on dispersion measurements. Knowledge of the growth mechanism and reaction conditions that affect morphology would be valuable in modifying the physical and electrical properties of metal oxide nanowires.

## Abstract 2

Tin oxide (SnO_2_) nanowires are produced by the calcination of tin glycolate (SnC_2_H_4_O_2_) nanowires, which are synthesized with tin oxalate (SnC_2_O_4_) and ethylene glycol *via* the so-called polyol process.

## Graphical abstract

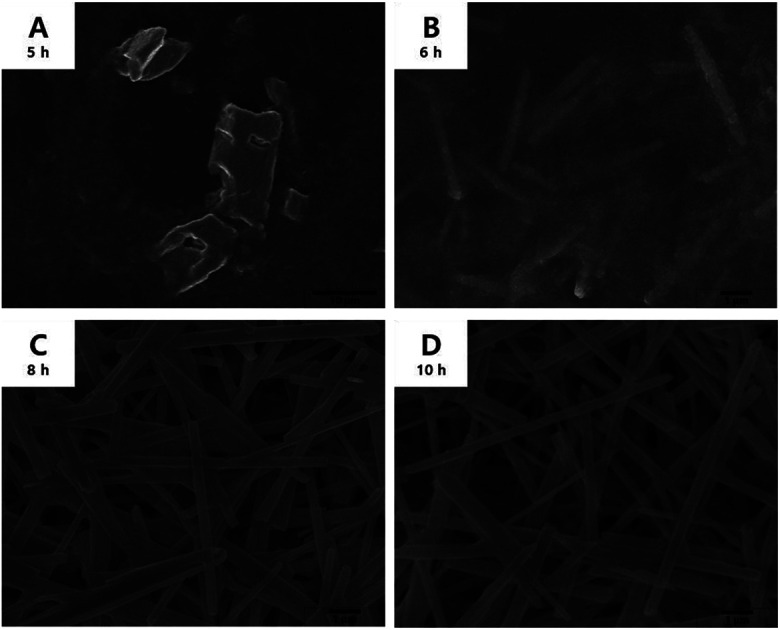

## 1. Introduction

SnO_2_-based nanomaterials have been widely applied as n-type semiconductor gas sensors for sensing reductive gases (*E*_g_ = 3.64 eV at 300 K),^1–7^ transparent conducting oxides in optoelectronic devices, thin film solar energy cells, and anode materials in high-capacity lithium-ion batteries.^8–16^ In particular, high-performance, flexible, and stretchable nanomaterial-based devices can be realized by using SnO_2_ nanowires.^17–19^ Therefore, the easy and large-scale synthesis of SnO_2_ nanowires remains an attractive field of research.

SnO_2_ nanowires have been synthesized mainly through a vapor–solid or vapor–liquid–solid process.^18,20–23^ The disadvantages of these methods are the high temperatures required for synthesis, costly scale-up, and difficulty in synthesizing a reproducible and uniform nanowire. In recent years, the so-called polyol process, which involves the use of ethylene glycol (EG), has been proposed to overcome these disadvantages.^24^ EG has been widely used for the synthesis of metal nanomaterials because of its relatively high boiling point and strong reducing power. In particular, EG has been used as a cross-linking reagent to synthesize various metal nanowires.^25–28^

Jiang *et al.* successfully synthesized a polycrystalline SnO_2_ nanowire using this process.^25,29^ The SnO_2_ nanowire was mainly applied as a gas sensor and exhibited excellent performance.^30^ Although the polyol process is simple and easy to scale up, using it to directly apply the as-synthesized nanowire to a device is difficult. Because of the difficulty in controlling the size and morphology of the nanowires, there have been few in-depth investigations of this method.

The mechanism of SnO_2_ nanowire synthesis proposed by Wang *et al.* involves the gradual replacement of tin oxalate (SnC_2_O_4_) by EG to form tin glycolate, which oligomerizes into long chains that assemble into ordered bundles.^31^ The SnO_2_ nanowire was produced by the heat treatment of the tin glycolate nanowire. However, there has been no proposed mechanism to suggest the possibility of controlling the size and morphology of the SnO_2_ nanowire during the polyol process, which would allow the control of its physical and electrical properties. Therefore, scanning electron microscopy (SEM), transmission electron microscopy (TEM), ^1^H NMR, ^13^C NMR, Fourier-transform IR (FTIR), high-performance liquid chromatography (HPLC), and thermogravimetric analysis (TGA) were performed for a detailed study on the mechanism of tin glycolate nanowire synthesis *via* the polyol process. Morphological changes in the SnO_2_ nanowire, and the resulting increase in dispersibility, were also investigated to validate the proposed mechanism.

## 2. Experimental section

### 2.1 Chemicals and materials

Tin(ii) oxalate, EG, 1,2-propanediol (PG), sulfuric acid, hydrochloric acid, and ethanol were purchased from Sigma-Aldrich. Deuterated dimethyl sulfoxide (DMSO-d6) for NMR analysis was purchased from Cambridge Isotope Laboratories. All reagents were used as received without further purification. The carbon-coated copper grids used in the TEM analysis were purchased from Ted Pella, Inc.

### 2.2 Instrumentation

SEM was performed on a JEM-7610F Schottky field emission scanning electron microscope (JEOL, Ltd.) operated at an accelerating voltage of 5.0 kV, and TEM was conducted on the JEM-2100F field emission electron microscope (JEOL, Ltd.) operated at an accelerating voltage of 200 kV. The SEM and TEM samples were prepared by dropping the ethanol suspension onto the copper grid and drying it in a vacuum desiccator. HPLC was performed on an Agilent 1260 HPLC system (Agilent Technologies) equipped with an Aminex HPX-87H column (Bio-Rad) and a refractive index detector, using 5.0 mM H_2_SO_4_ as the eluent. FTIR spectra were measured in the attenuated total reflection mode using a Nicolet iS50 FTIR spectrometer (Thermo Fisher Scientific). ^1^H NMR and ^13^C NMR (400 MHz) spectra were recorded using an AVANCE III NMR spectrometer (Bruker). % transmittance was measured using an S-3100 UV-visible scanning spectrophotometer (SCINCO). X-ray diffraction (XRD) data were measured using a powder type sample on a D/Max 2500V X-ray diffractometer (Rigaku). TGA curves were measured using a DSC 2010/SDT 2960 analyzer (TA Instruments) at a rate of 10 °C min^−1^ under N_2_ gas. Elemental analysis (EA) was conducted on a FlashEA 11.

### 2.3 Preparation of tin glycolate nanowires

Preparation of tin glycolate was conducted using a modification of a previously described method.^31^ Briefly, 1.00 g of tin oxalate was stirred vigorously into 100 mL of EG and heated to 140 °C. After 12 h, the resulting solution was cooled to room temperature. The white precipitate was separated from the solution using a centrifuge and washed with ethanol to remove the residual EG.

### 2.4 Preparation of the branched tin glycolate nanowires

A mixture of 1.00 g of tin oxalate, 90 mL of EG, and 10 mL of PG was stirred vigorously at 140 °C. The subsequent procedure is the same as in the preparation of tin glycolate nanowires.

## 3. Results and discussion

As mentioned before, the growth mechanism of the tin glycolate nanowires proposed by Jiang *et al.* was that the oxalate bonded to tin is replaced by EG to form a chain-like complex.^31^ However, the authors did not report the detailed mechanism of how the deprotonated EG coordinates to tin and produces the nanowires. This mechanism was determined in this study by analyzing the precipitate and solution formed during the reaction.

**Fig. 1:**
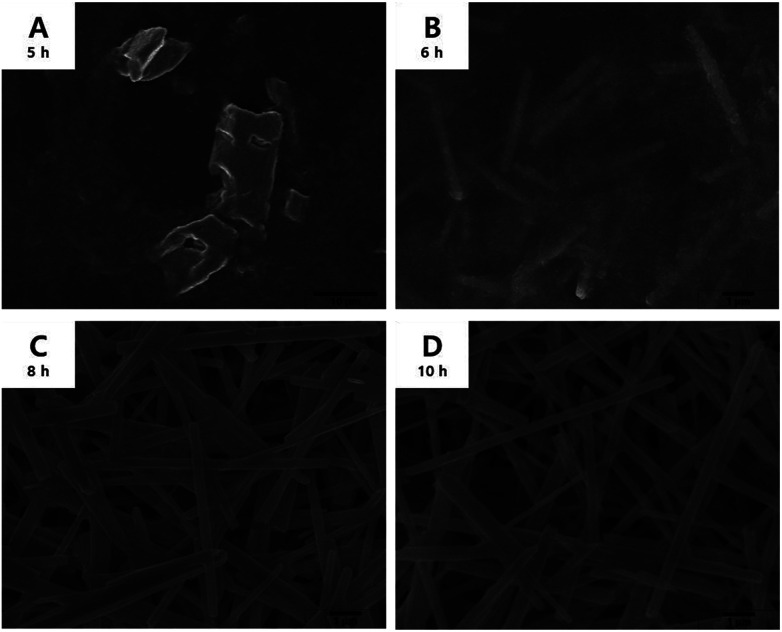
SEM images of the precipitate over time. (A) Before complete dissolution of tin oxalate; (B) *L* = 1.85 μm, *R* = 0.37 μm, aspect ratio = 10; (C) *L* = 6.95, *R* = 0.37, aspect ratio = 37.6; (D) *L* = 9.25, *R* = 0.37, aspect ratio = 50.

Tin oxalate began to slowly dissolve in EG at 140 °C and became a clear solution after 5 h. A white precipitate began to form slowly after 6 h, resulting in a milk-like solution after 8 h. Fig. 1 shows the SEM images of the precipitate in solution during the reaction. In particular, Fig. 1A and B show that nanowires begin to form after 5 h when tin oxalate has completely dissolved in EG. The thickness of the nanowires was about 0.37 μm, and it does not change after 10 h of reaction. Moreover, the length gradually increased with time at a rate of 1.85 μm h^−1^ (Fig. S1†). This means that the nanowire only grows in the longitudinal direction and not in both directions. The final synthesized nanowire was relatively thick (0.37 μm) and had a length of 9.25 μm (aspect ratio = 50).

FTIR spectroscopy was performed to confirm the functional groups of the precipitate. The spectra show that the precipitate was tin oxalate until 5 h after the reaction but became tin glycolate after 6 h (Fig. S2†). Particularly, the –OH bond peak at around 3400 cm^−1^ is not observed after 6 h of reaction.^32,33^ This indicates that after tin oxalate was completely dissolved in EG, and it was glycolate, not EG, that is coordinated to tin(ii). TGA analysis was performed to determine the mass ratio of tin(ii) to glycolate (Fig. S3†). There was a 33% mass reduction at around 300 °C due to glycolate, and the ratio of tin to glycolate was 1 : 1 during the weight loss. The remaining mass decreased to 64% up to 500 °C, after which it did not change. Elemental analysis of tin(ii) glycolate showed 12.57% C and 2.59% H, confirming that tin(ii) and glycolate were coordinated at a 1 : 1 ratio (calc. C: 13.44, H: 2.26).

The composition of the reactants and changes in the constituents were confirmed by ^1^H NMR and ^13^C NMR analyses (Fig. S4†). The oxalate ion did not contain hydrogen atoms; hence, there should be no proton peak in the ^1^H NMR spectra in the DMSO-d6 solvent. Nevertheless, a peak at 8.19 ppm is observed. In the ^13^C NMR spectra, a peak at 158 ppm corresponding to oxalate and an additional peak at 163 ppm are observed. The additional peaks in the ^1^H NMR and ^13^C NMR spectra are due to formic acid.^34–36^ The composition of the reaction solution was investigated by HPLC (Fig. S5†). Fig. S6† shows that the ratio of oxalate and formic acid remained constant until the 5^th^ hour, after which the concentration of oxalate decreased and that of formic acid increased sharply.

**Scheme 1:**
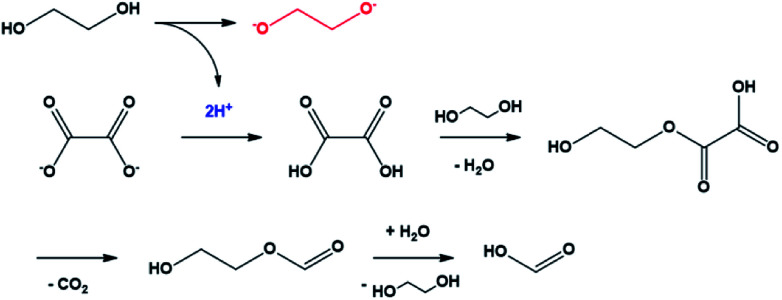
Mechanism of oxalate conversion to formic acid initiated by ethylene glycol deprotonation.

The mechanism of the conversion of oxalic acid to formic acid in glycerol, which has a structure similar to that of EG, was well known.^37,38^ In this reaction, two protons were required for a series of reactions that could convert oxalate to oxalic acid and, subsequently, to formic acid. In the case of EG, it can be easily predicted that the compound acted as the proton donor. In other words, the conversion of oxalate to formic acid was the driving force for the conversion of EG to ethylene glycolate (Scheme 1). The ethylene glycolate formed was nucleophilic and could easily bond to tin(ii). Owing to the presence of two alkoxide groups, glycolate acted as a complexation agent to form the tin glycolate nanowire.

In summary, FTIR analysis indicated that there was no –OH bond in the as-synthesized tin glycolate, and TGA and elemental analyses showed that tin and glycolate were coordinated at a 1 : 1 ratio. The SEM images showed that the nanowire grew only in the longitudinal direction and not in the transverse direction; the structure elongated as tin glycolate molecules formed O–Sn–O bonds. Thus, the structure formed became bundle-shaped owing to the van der Waals interactions and resembled a nanowire.

To validate our proposed mechanism and control the size and morphology of the nanowire, tin glycolate was synthesized by mixing EG and PG. A small amount of PG is added so that its methyl group will interfere with the growth of the tin glycolate nanowire bundle. This occurs when the *cis* form of the hydroxyl groups of PG is more stable than the *trans* form.

**Fig. 2:**
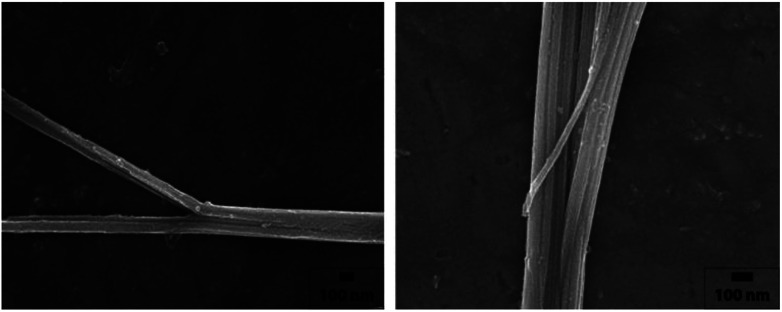
SEM images of tin glycolate nanowire synthesized with EG and PG.

Fig. 2 shows the SEM images of the tin glycolate nanowire synthesized by this method. The as-synthesized tin glycolate is observed not in a complete nanowire morphology but in a branched one. The average thickness of the branched nanowire was 0.43 μm, and the average length was 11.0 μm; thus, the nanowire was slightly thicker and longer than the one synthesized with pure EG. The nanowire was dissolved in acid to confirm the composition ratio of EG and PG by ^1^H NMR (Fig. S7†). The peak at 0.55 ppm corresponding to the PG methyl group and the peak at 3.09 ppm corresponding to EG were integrated and confirmed to have compositions of 5.00 and 100, respectively. Thus, a small amount of PG was incorporated into the tin glycolate nanowire and changed its shape.

No bundles were observed directly when the nanowires were synthesized using only EG; however, SEM images of the tin glycolate nanowire synthesized by mixing EG and PG show that each wire is bundled (Fig. 2). Particularly, a few bundles are divided and a branch-like shape is observed. For the branched nanowire, the wire thickness before division and the sum of the thicknesses of the divided wires were similar. These results indicate that as the nanowire forms, the thickness and length do not increase simultaneously, only the latter.

**Fig. 3:**
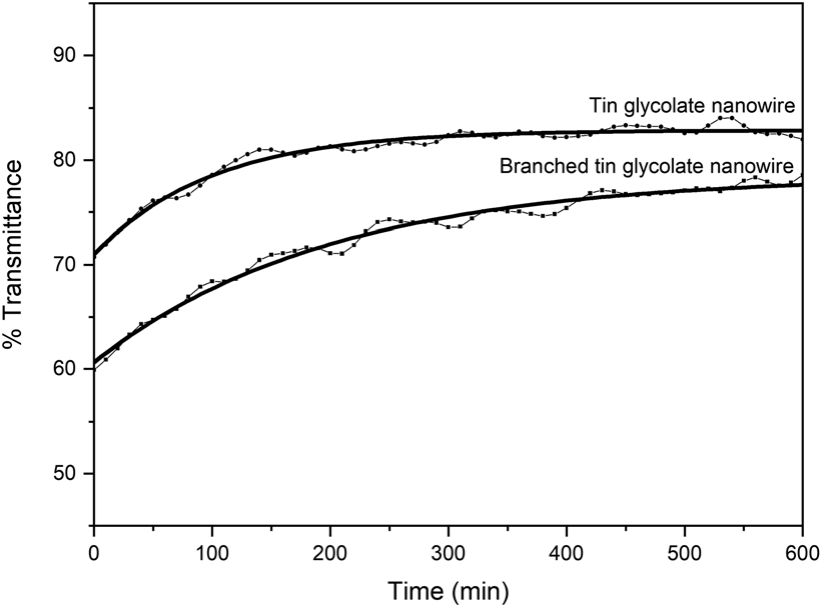
% transmittance over time of tin glycolate nanowires (0.01 g mL^−1^) dispersed in ethanol.

The branched nanowires were expected to form a strong network owing to increased contact between the wires caused by the reduction in wire thickness and a concomitant increase in the surface area. The stronger network would result in more efficient nanodevices. Therefore, the dispersibility was measured by dispersing the nanowires synthesized *via* the two methods in ethanol. The dispersibility was taken as the change in %transmittance over time (Fig. 3). This means that if the dispersibility is good, the permeability will increase gradually; however, if the dispersibility is poor, the permeability will increase rapidly. Initially, the nanowires synthesized with EG and PG showed a transmittance about 10% lower than that of the nanowires synthesized with only EG. The low transmittance is due to the larger surface area of the branched nanowires. The %transmittance of the nanowires synthesized with EG and PG require about 600 min to reach equilibrium, while those synthesized with only EG reach equilibrium within 300 min. That is, the branched nanowires have better dispersion and larger surface area than unbranched nanowires.

The morphologies, aspect ratios, and specific surface areas of tin glycolate nanowires synthesized using various concentrations of tin oxalate (1.0 g, 2.0 g, 10.0 g with 100 mL of EG) are given in Fig. S8.† The thickness of the tin glycolate nanowires generally increased as the concentration of tin oxalate increased. The tin glycolate powder would release rapidly when the tin oxalate concentration was high. This increased the size of the tin oxalate powder particles and led to thick nanowires due to the anisotropic polymerization in the longitudinal direction.

As previously reported, polycrystalline rutile-phase tin oxide nanowires were synthesized without morphology change by annealing the tin glycolate nanowire at 400 °C for 3 h (Fig. S9†).^25,31^ This suggests that the aspect ratio of the tin oxide nanowire can be controlled by controlling the concentration of tin oxalate. The tin oxide nanowire can be easily applied to various nanodevices because it is easily dispersed by sonication for 10 min in ethanol or water.

## 4. Conclusions

Tin glycolate nanowires were synthesized using tin oxalate and EG. The SEM images confirmed that the nanowires only grew in the longitudinal direction, and only tin glycolate, not EG or oxalate, was observed by FTIR spectroscopy. ^1^H NMR, ^13^C NMR, and HPLC analyses of the reaction solution revealed that oxalate changed to formic acid. This reaction required two protons, and EG acted as the proton donor and produced glycolate. Therefore, glycolate is nucleophilic and can be easily coordinated to the tin ion to form tin glycolate.

The tin glycolate nanowire was also synthesized using a mixture of EG and PG to confirm that the nanowire morphology was due to the O–Sn–O bonding of the tin glycolate molecules. A branched nanowire was formed by this method owing to steric hindrance by the methyl group of PG. The similar thickness before and after branching confirmed that the nanowire did not become thicker but only longer during synthesis. The branched nanowire was also confirmed to have excellent dispersibility compared to the bundled nanowire based on the % transmittance.

The polyol process has been applied for the fabrication of various nanodevices because of their easy mass production. However, if the mechanism of this process is accurately known, metal oxide nanowires can be easily synthesized using various metal salts. Therefore, this study proposed a mechanism for tin glycolate nanowire synthesis *via* the polyol process and applied this knowledge to the synthesis of a branched nanowire.

## Conflicts of interest

There are no conflicts to declare.

## Supplementary Material

RA-009-C8RA09738K-s001
