# Supplementary material for: Growth mechanism of SnC2H4O2 nanowires prepared by the polyol process as SnO2 precursor nanowires
Source: RSC Adv. 2019 Jan 23;9(6):3203–7. doi: 10.1039/c8ra09738k (PMC9060261; doi:10.1039/c8ra09738k)
Supplement: RA-009-C8RA09738K-s001 [file RA-009-C8RA09738K-s001.pdf]

Electronic Supplementary Material (ESI) for RSC Advances.
This journal is © The Royal Society of Chemistry 2019

Supplementary information

Fig. S1. SEM images of precipitate over time.

Fig. S2. FTIR spectra of precipitate over time.

Fig. S3. TGA curve of tin glycolate nanowire.

Fig. S4. ^{1}H NMR and ^{13}C NMR spectra of solution over time.

Fig. S5. HPLC chromatograms of solution over time.

Fig. S6. Constituent ratio of oxalic acid and formic acid.

Fig. S7. ^{1}H NMR spectra of tin glycolate nanowire synthesized with EG and PG.

Fig. S8. TEM images of tin glycolate nanowire and sample specifications.

Fig. S9. (A) TEM image and (B) XRD pattern of SnO_2 nanowire annealed at 400℃ for 3 h.

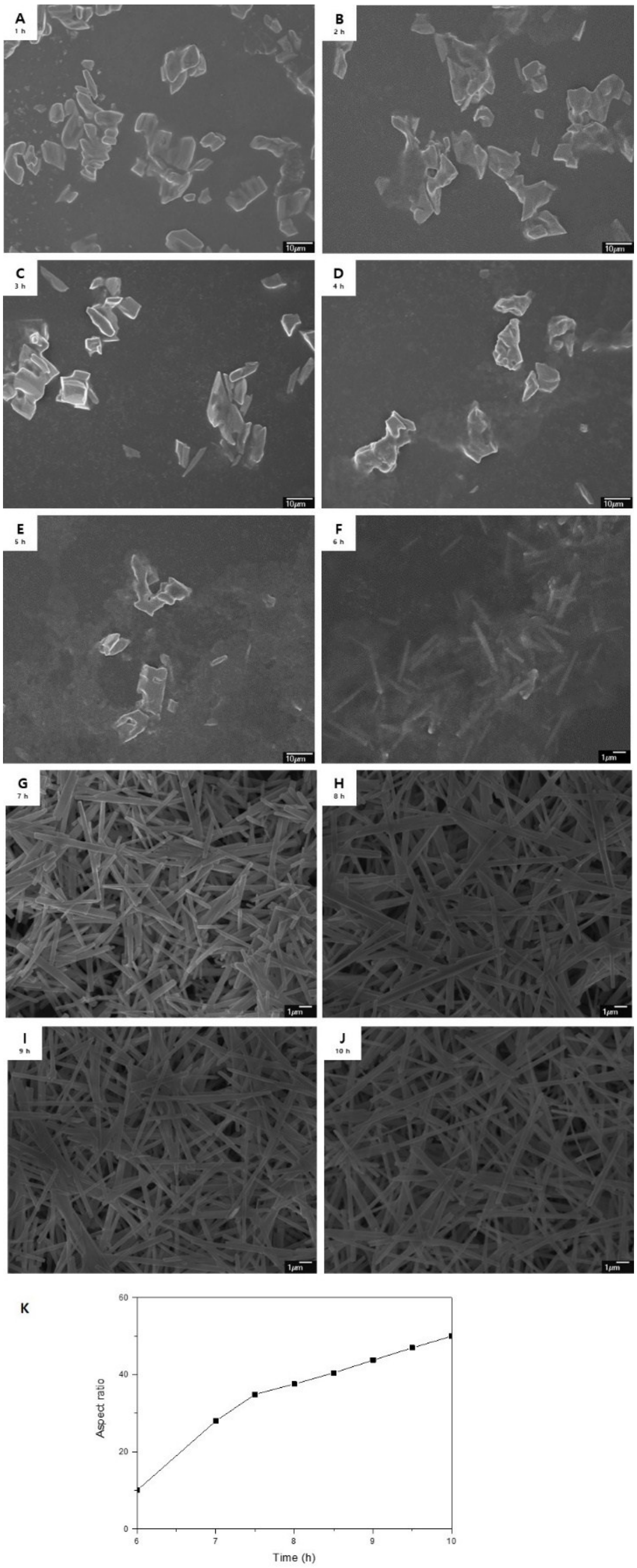

Fig. S1. SEM images of precipitate over time. (A), (B), (C), (D), and (E) Before complete dissolution of tin oxalate; (F) L = 1.85 μm, R = 0.37 μm, Aspect ratio = 10; (G) L = 5.10, R = 0.36, Aspect ratio = 28; (H) L = 6.95, R = 0.37, Aspect ratio = 37.6; (I) L = 8.1, R = 0.37, Aspect ratio = 43.8; (J) L = 9.25, R = 0.37, Aspect ratio = 50; (K) Time dependence of aspect ratio.

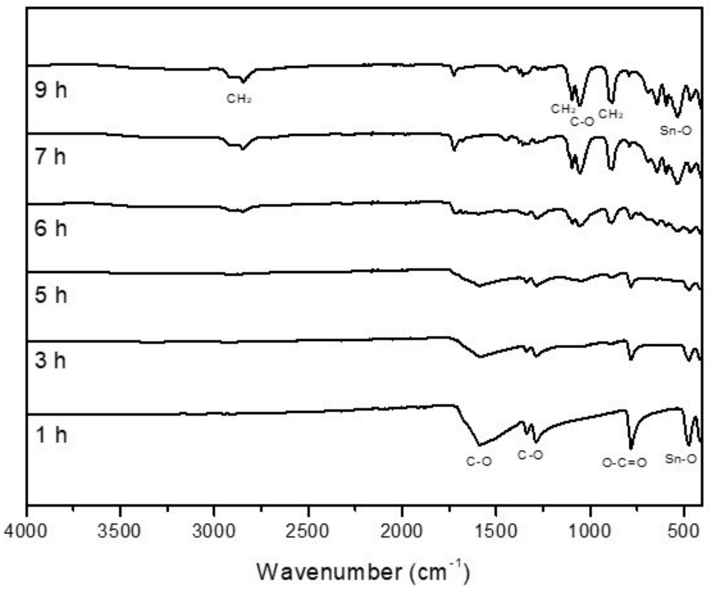

Fig. S2. FTIR spectra of precipitate over time.

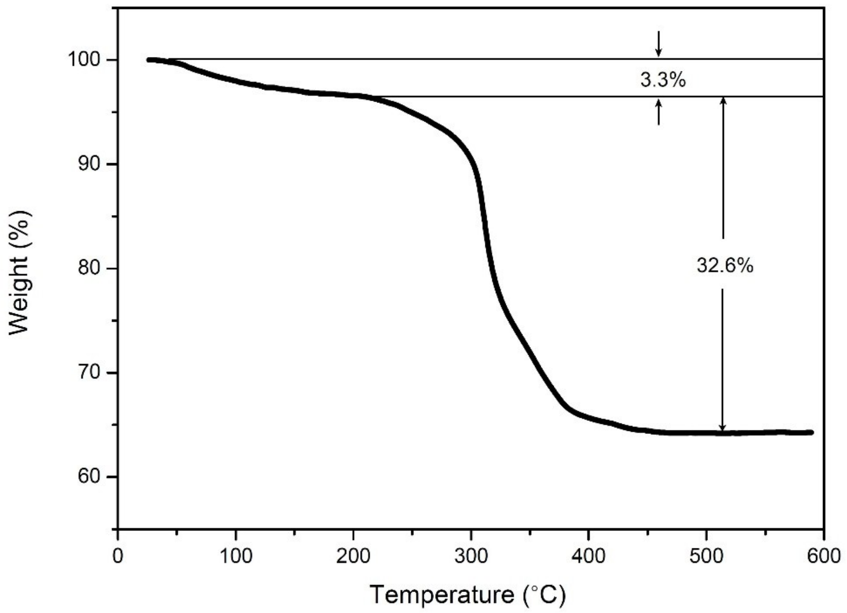

Fig. S3. TGA curve of tin glycolate nanowire.

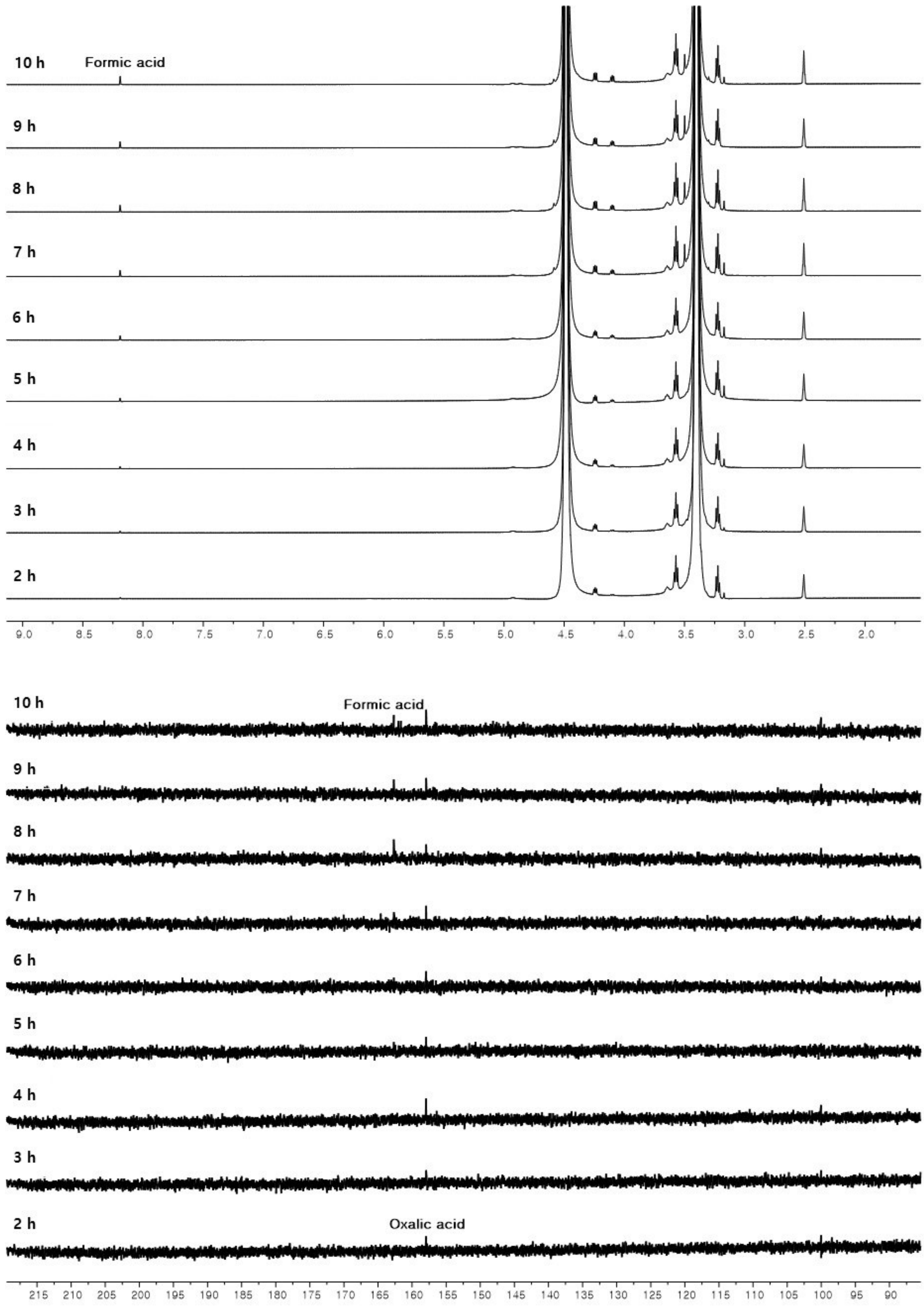

Fig. S4. ^{1}H NMR (top) and ^{13}C NMR (bottom) spectra of solution over time.

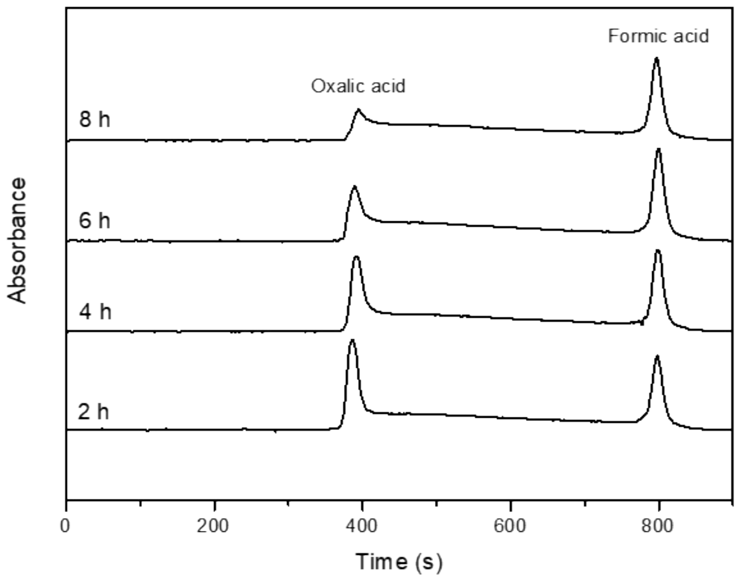

Fig. S5. HPLC chromatograms of solution over time.

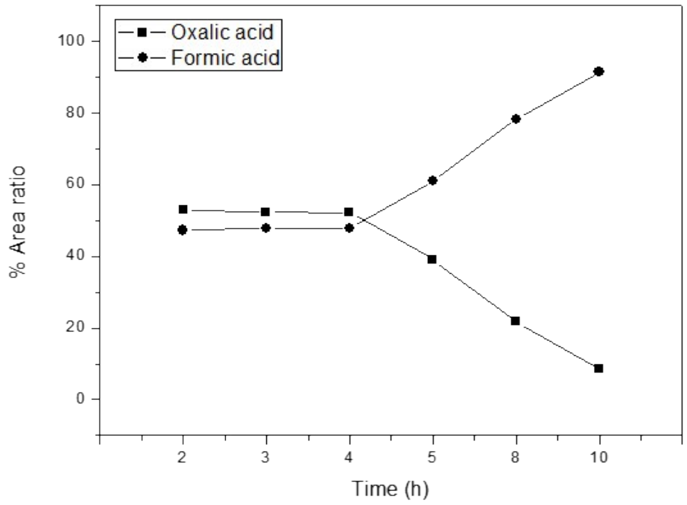

Fig. S6. Constituent ratio of oxalic acid and formic acid.

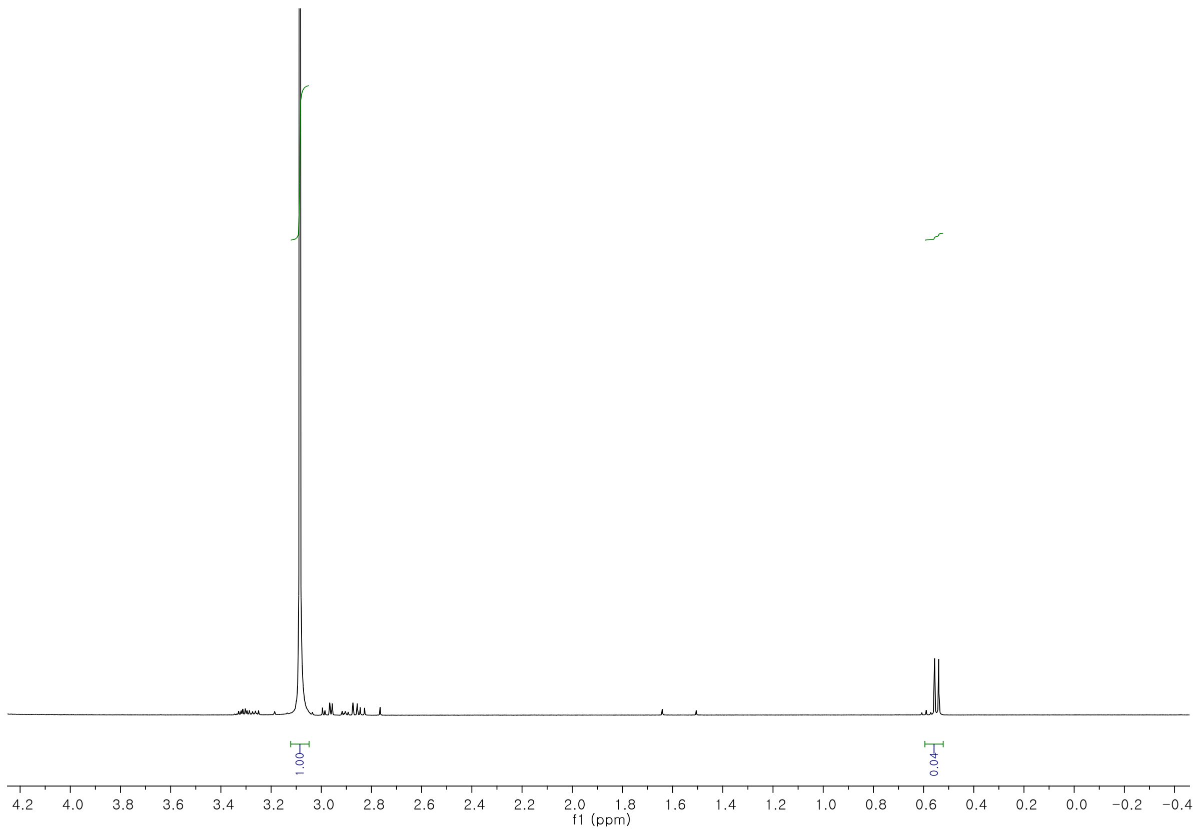

Fig. S7. ^{1}H NMR spectra of tin glycolate nanowire synthesized with EG and PG.

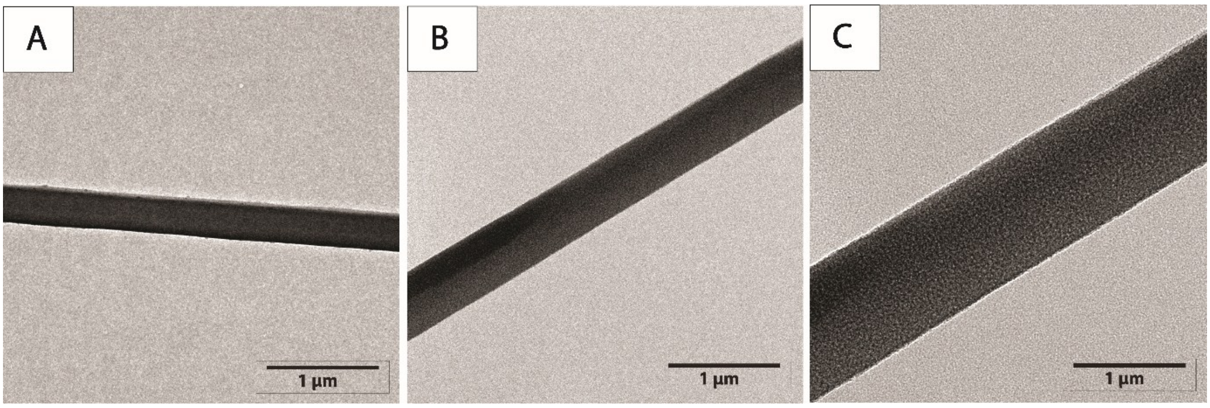

Tin glycolate nanowire	Tin oxalate (g) to 100 mL EG	Thickness (μm)
A	1.0	0.37
B	2.0	0.57
C	10.0	1.11

Fig. S8. TEM images of tin glycolate nanowire and sample specifications.

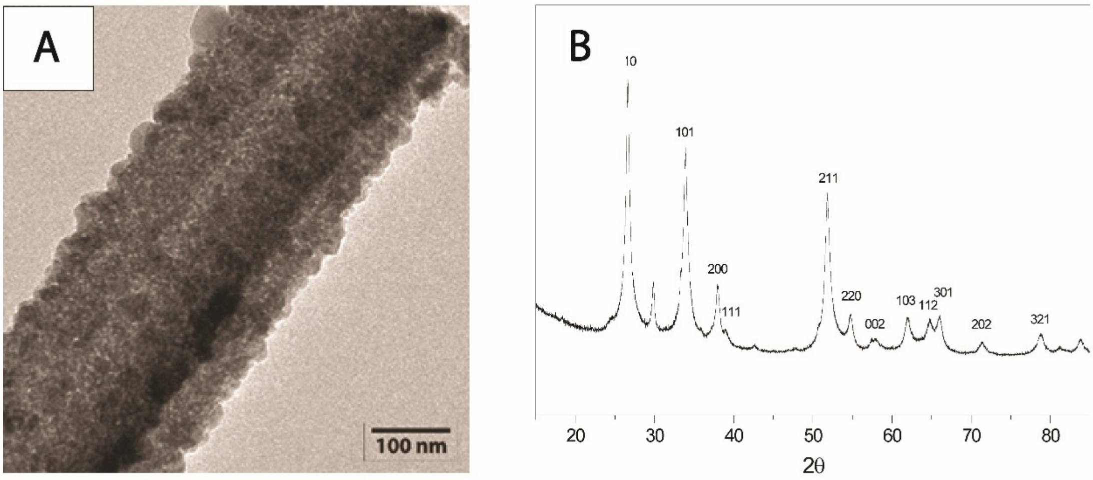

Fig. S9. (A) TEM image and (B) XRD pattern of SnO_2 nanowire annealed at 400℃ for 3 h.
